# Human Herpesvirus 6 Associated With a Secondary Central Nervous System Vasculitis in an Immunocompetent Adult: A Case Report

**DOI:** 10.7759/cureus.88305

**Published:** 2025-07-19

**Authors:** Olivia Feuchter Ruy Sánchez, Rubén A Almela Mendoza, Elisa Bribiesca Contreras

**Affiliations:** 1 Internal Medicine, Instituto Mexicano del Seguro Social, Chihuahua, MEX; 2 Neurology, Instituto Mexicano del Seguro Social, Chihuahua, MEX

**Keywords:** atypical neurological presentation, cerebrospinal fluid analysis, cns vasculitis, encephalitis, human herpesvirus 6, immunocompetent host, neuroimaging, pcr diagnostics, viral vasculopathy

## Abstract

Human herpesvirus 6 (HHV-6) encephalitis typically affects children under three years of age, and adult cases are rare, usually occurring in immunocompromised individuals such as hematopoietic stem cell transplant recipients. HHV-6 can establish latency and reactivate under conditions of immunosuppression. Several neurotropic viruses, including varicella-zoster virus and herpes simplex virus, are known to induce central nervous system (CNS) vasculitis, but HHV-6 has not been clearly associated with this complication. We report the case of a 46-year-old immunocompetent woman presenting with encephalopathy, hemiparesis, and cerebellar signs. Brain MRI revealed ischemic lesions in multiple vascular territories and imaging features suggestive of cerebral vasculitis. Cerebrospinal fluid polymerase chain reaction (CSF PCR) was positive for HHV-6. Other etiologies, including neoplastic and autoimmune causes, were ruled out. The patient was treated with ganciclovir and dexamethasone at doses appropriate for CNS vasculitis. This case highlights a rare presentation of HHV-6-associated CNS vasculitis in an immunocompetent host and emphasizes the need to consider viral etiologies in the differential diagnosis of CNS vasculopathies.

## Introduction

Human herpesvirus 6 (HHV-6) is a neurotropic virus that commonly causes exanthem subitum (roseola infantum) in early childhood and establishes lifelong latency in various tissues, including the central nervous system (CNS). Reactivation of latent HHV-6 is well documented in immunocompromised individuals, particularly following hematopoietic stem cell transplantation, and may result in severe complications such as encephalitis [1-3]. However, CNS manifestations of HHV-6 in immunocompetent individuals are rare and not fully understood [4].

Among the various neurological complications associated with viral infections, CNS vasculitis is most frequently linked to varicella-zoster virus (VZV) and herpes simplex virus (HSV), while HHV-6 has not been conclusively associated with this condition [2].

Here, we present the case of a 46-year-old immunocompetent woman with encephalopathy and focal neurological deficits secondary to HHV-6-associated CNS vasculitis. This case adds to the growing body of literature suggesting that HHV-6 reactivation may present with atypical and severe neurological syndromes even in immunocompetent hosts [4]. It also underscores the diagnostic value of molecular techniques and advanced neuroimaging in identifying treatable causes of CNS vasculopathies.

## Case presentation

A previously healthy 46-year-old immunocompetent woman presented with a two-week history of progressive neurological symptoms, including severe headache, dysarthria, memory impairment, executive dysfunction, psychomotor agitation, and progressive left-sided weakness limiting ambulation. On admission, she was disoriented, with left hemiparesis and right-sided cerebellar signs, notably hemiataxia. No fever or signs of systemic infection were present.

The patient had no relevant past medical history, comorbidities, or chronic medication use. There was no history of prior neurological or psychiatric conditions.

**Figure 1:**
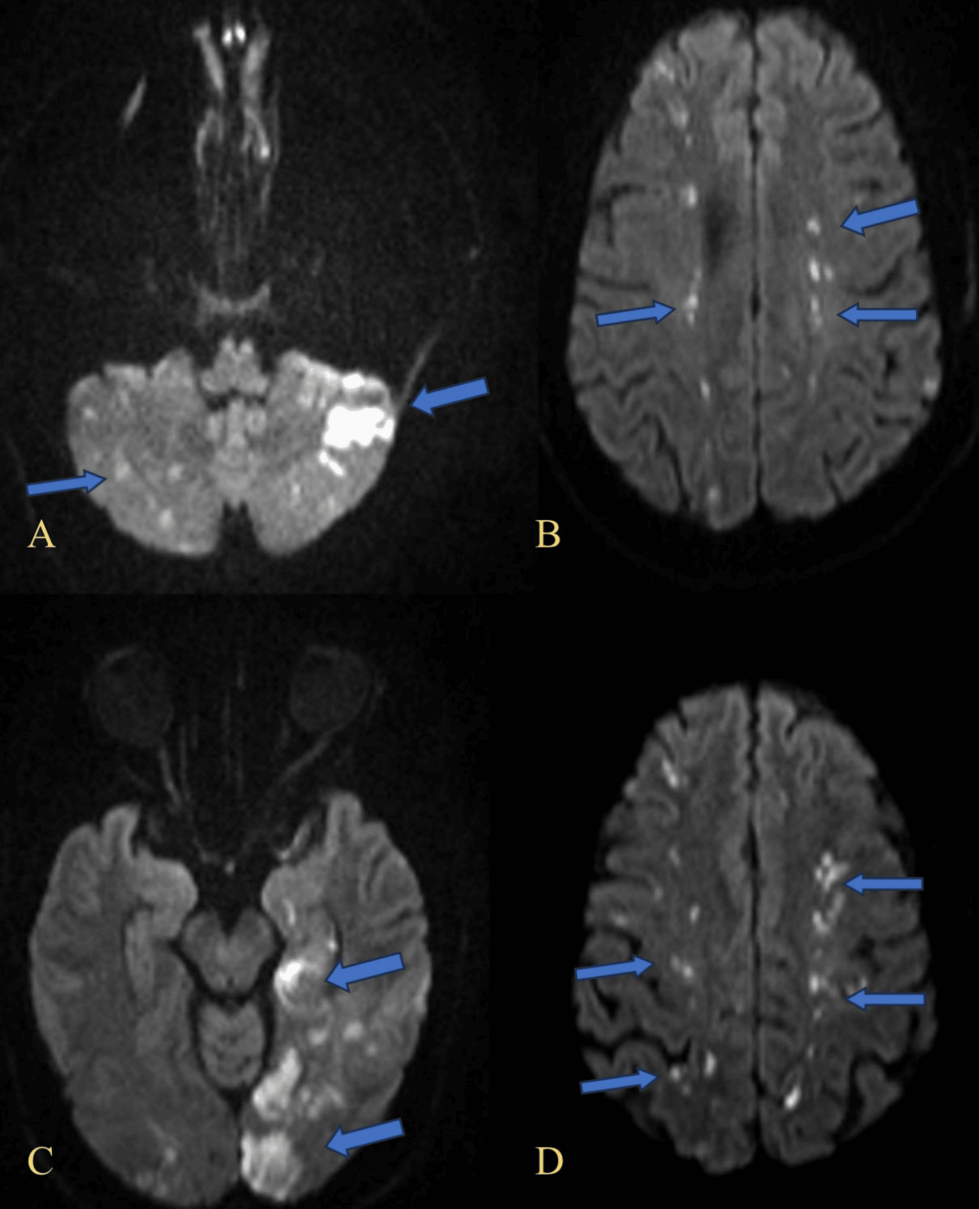
Diffusion-weighted imaging (DWI) demonstrating multifocal areas of restricted diffusion. (A) Bilateral hyperintensities in the cerebellar hemispheres, more prominent on the left (blue arrows), compatible with acute ischemia. (B) Multiple foci of restricted diffusion in the bilateral subcortical white matter of the frontal lobes (blue arrows), indicative of acute embolic infarcts. (C) Hyperintense lesions in the left occipital lobe (blue arrows), consistent with acute ischemic injury. (D) Scattered foci of restricted diffusion in the bilateral parietal cortices (blue arrows), suggestive of multiple acute cortical infarctions. DWI: diffusion-weighted imaging

As shown in Figure 1A-1D, diffusion-weighted imaging (DWI) revealed multiple hyperintense foci suggestive of acute ischemic lesions in various vascular territories.

**Figure 2:**
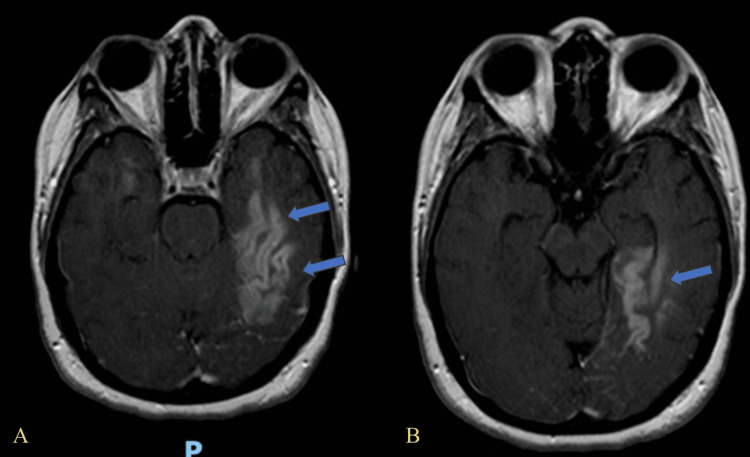
Axial post-contrast T1-weighted MRI showing cortical and subcortical enhancement in the left temporal lobe. (A) Hyperintense signal involving the lateral aspect of the left temporal lobe cortex (blue arrows), consistent with cortical enhancement and encephalitic changes. (B) More medial axial slice showing confluent enhancement in the left mesial temporal lobe, including the hippocampus (blue arrow).

Cortical enhancement in the left temporal lobe is demonstrated in Figure 2A and Figure 2B, evident in the post-contrast axial MRI.

**Figure 3:**
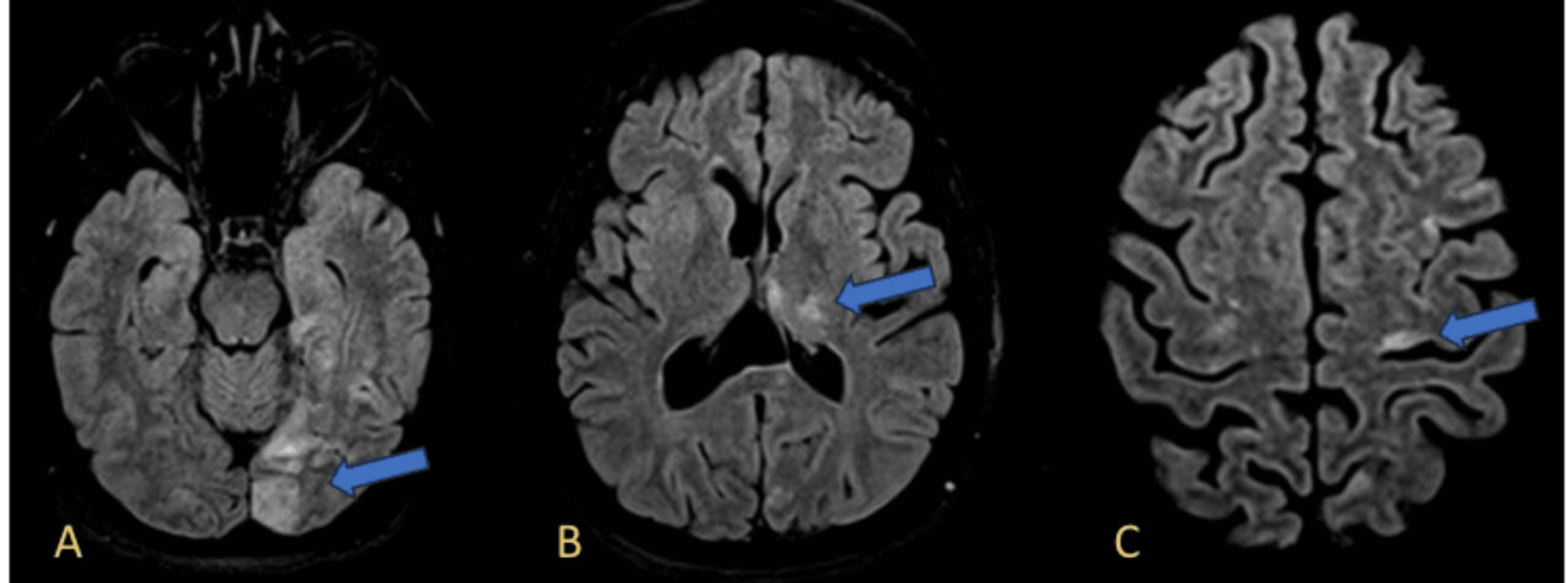
FLAIR sequences showing multifocal hyperintensities. (A) Hyperintense signal in the left occipital lobe (blue arrow), suggestive of acute infarction. (B) Restricted diffusion in the left thalamus (blue arrow), consistent with subcortical ischemia. (C) Cortical hyperintensity in the left frontal lobe, suggestive of acute cortical ischemia (blue arrow). FLAIR: fluid-attenuated inversion recovery

Brain MRI with contrast demonstrated multifocal T2 and FLAIR hyperintensities involving the left temporal lobe, left parieto-occipital region, bilateral frontal cortices, and cerebellar hemispheres. Figure 3A-3C illustrates these findings, highlighting the distribution of cortical and deep white matter lesions.

**Figure 4:**
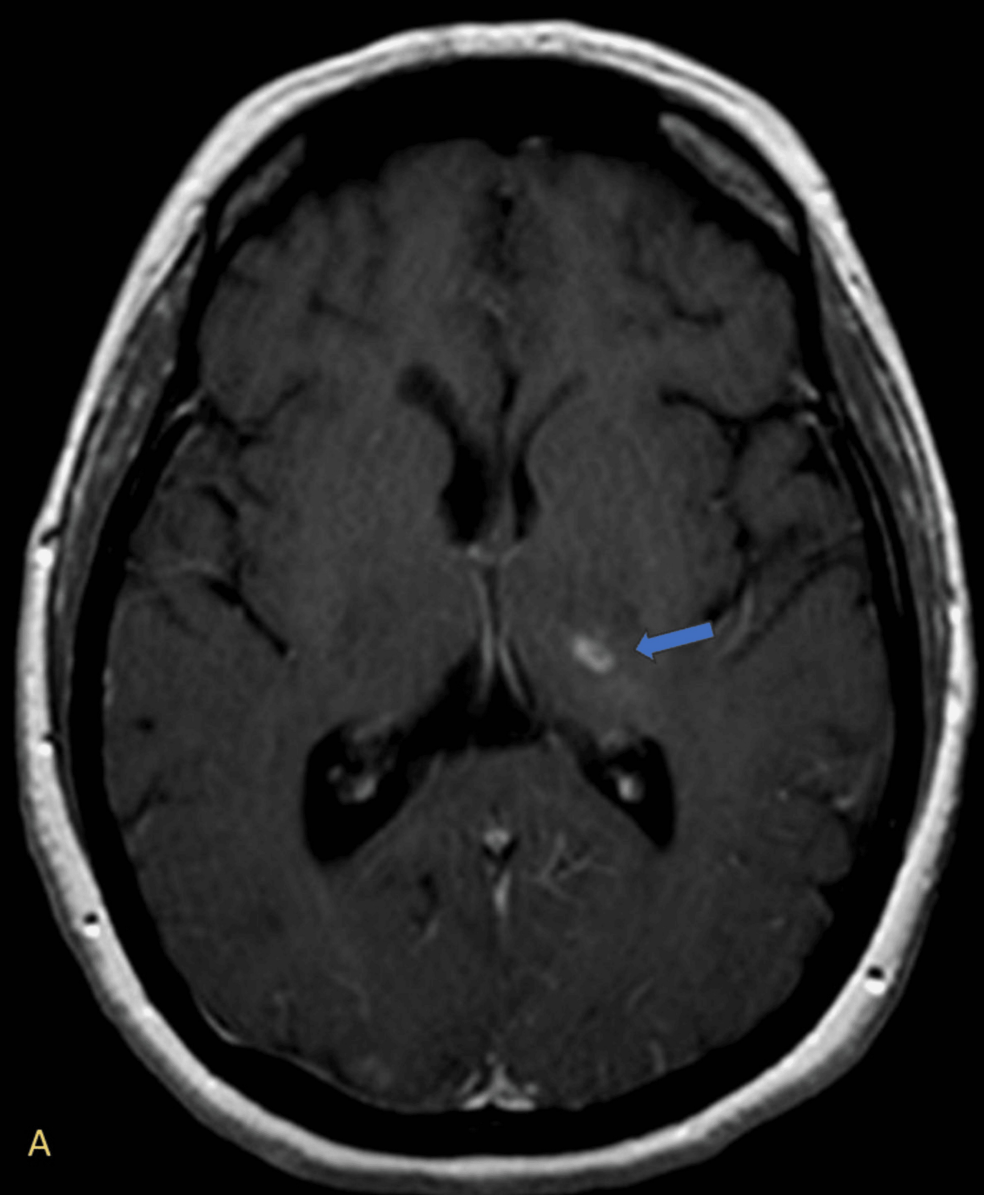
Axial post-contrast T1-weighted MRI showing a ring-enhancing lesion in the left thalamus. (A) A small ring-enhancing lesion is noted in the left thalamus (blue arrow), characterized by peripheral contrast enhancement with a hypointense center. This radiologic pattern is suggestive of a ring-enhancing lesion, typically seen in infectious processes.

Post-contrast imaging revealed ring-enhancing lesions in the left thalamus, suggestive of an infectious vasculitic process (Figure 4).

**Figure 5:**
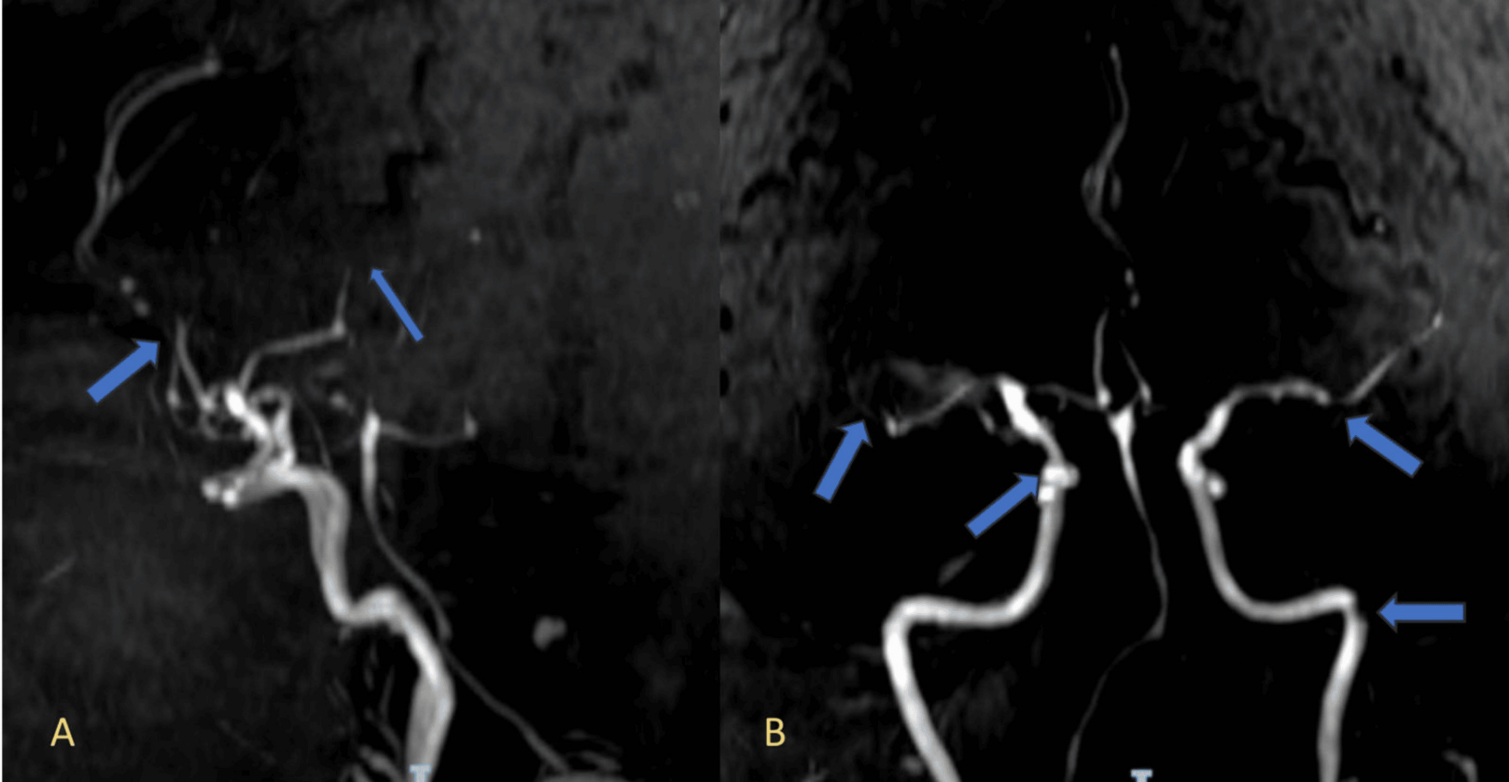
Magnetic resonance angiography (MRA) showing segmental narrowing of intracranial vessels. (A) MRA image reveals multifocal areas of segmental narrowing in the anterior cerebral artery (ACA) and middle cerebral artery (MCA) territories (blue arrows), suggestive of vasculitic changes. (B) Additional areas of irregular caliber and narrowing are observed in both anterior circulations and the basilar artery (blue arrows), supporting the presence of a diffuse vasculopathy.

Three-dimensional time-of-flight (3D TOF) magnetic resonance angiography showed segmental narrowing of several intracranial vessels, as illustrated in Figure 5A and Figure 5B, which depicts anterior circulation vessel narrowing.

Lumbar puncture showed clear cerebrospinal fluid with normal glucose, protein, and white cell count. polymerase chain reaction (PCR) analysis identified HHV-6 DNA, confirming viral involvement. Extensive evaluation for other infectious, neoplastic, and autoimmune etiologies was performed. These included testing for HIV, hepatitis B and C, VDRL, ANA, ANCA, rheumatoid factor, and antiphospholipid antibodies, all of which were negative. Workup for autoimmune encephalitis (including anti-NMDA and other neuronal antibodies) and vasculitis panels also yielded negative results. Digital subtraction angiography (DSA), the gold standard for CNS vasculitis, was not performed due to a lack of availability at our center. However, magnetic resonance angiography (MRA) was used as an alternative and showed multifocal segmental narrowing in the anterior and posterior circulations, supporting the diagnosis of vasculitis. 

The patient was started on intravenous ganciclovir (5 mg/kg every 12 hours) and dexamethasone, with a treatment course of 14 days. Her neurological status showed initial improvement, with resolution of agitation and partial recovery of motor and cognitive functions. However, despite these clinical gains, her condition deteriorated subsequently, and she unfortunately passed away during the same hospitalization.

## Discussion

This case highlights a rare presentation of CNS vasculitis secondary to HHV-6 infection in an immunocompetent individual. Vasculitis is a known complication of neurotropic viruses, particularly varicella-zoster virus (VZV) and herpes simplex virus types 1 and 2 (HSV-1, HSV-2) [1]. However, in a published cohort of HHV-6 encephalitis patients, vasculitis was not reported, underscoring the novelty of this association [2].

The imaging findings in our patient, which included multifocal ischemic lesions across various vascular territories and segmental vessel narrowing on magnetic resonance angiography, were consistent with vasculitis. The differential diagnosis included primary angiitis of the CNS, autoimmune encephalitis, and small-vessel vasculitis of other etiologies. However, these were considered less likely due to the absence of systemic involvement, negative autoimmune panels, and the detection of HHV-6 DNA in the cerebrospinal fluid (CSF). Additional evaluations excluded neoplastic causes.

Although HHV-6 reactivation typically occurs in immunocompromised states [1,3], our patient had no known immunosuppression, suggesting a possible atypical reactivation or a subclinical immune vulnerability. There are few reports of HHV-6-associated encephalitis in immunocompetent adults, but growing evidence supports this possibility [4]. Mekheal et al. reported a similar case in a young immunocompetent patient, confirming that HHV-6 can cause severe CNS involvement even in the absence of overt immunosuppression. However, none of these reports described concurrent vasculitis, further highlighting the uniqueness of the present case [4]. The patient received antiviral therapy with ganciclovir and corticosteroid treatment with dexamethasone at vasculitis-targeted doses; however, despite these interventions, the clinical course was unfavorable and ultimately led to the patient’s death.

While HHV-6 reactivation has been described in immunocompetent adults with encephalitic syndromes, to our knowledge, no previously published case has reported central nervous system vasculitis as a manifestation. This case expands the known clinical spectrum of HHV-6-associated neurological disease and emphasizes the importance of considering viral etiologies even in immunocompetent hosts.

Prompt diagnosis and appropriate management are essential, as viral-associated vasculitis may mimic other vascular, autoimmune, or neoplastic processes on imaging. Early recognition of viral etiologies allows for targeted antiviral therapy and may prevent unnecessary or inappropriate immunosuppressive treatment.

## Conclusions

HHV-6-associated vasculitis of the central nervous system is an extremely rare entity, particularly in immunocompetent patients. This case highlights the importance of including viral etiologies, including HHV-6, in the differential diagnosis of CNS vasculitis. Comprehensive neuroimaging and molecular diagnostics such as CSF PCR are essential tools for accurate diagnosis and guiding appropriate management. Greater clinical awareness of this possible association may lead to earlier recognition and improved outcomes.
